# Development and Application of Integrated Optical Sensors for Intense E-Field Measurement

**DOI:** 10.3390/s120811406

**Published:** 2012-08-21

**Authors:** Rong Zeng, Bo Wang, Ben Niu, Zhanqing Yu

**Affiliations:** State Key Lab of Power Systems, Department of Electrical Engineering, Tsinghua University, Beijing 100084, China; E-Mails: wang-b04@mails.tsinghua.edu.cn (B.W.); niuben@tsinghua.edu.cn (B.N.); yzq@tsinghua.edu.cn (Z.Y.)

**Keywords:** electric field, E-field sensor, Pockels effect, integrated optics

## Abstract

The measurement of intense E-fields is a fundamental need in various research areas. Integrated optical E-field sensors (IOESs) have important advantages and are potentially suitable for intense E-field detection. This paper comprehensively reviews the development and applications of several types of IOESs over the last 30 years, including the Mach-Zehnder interferometer (MZI), coupler interferometer (CI) and common path interferometer (CPI). The features of the different types of IOESs are compared, showing that the MZI has higher sensitivity, the CI has a controllable optical bias, and the CPI has better temperature stability. More specifically, the improvement work of applying IOESs to intense E-field measurement is illustrated. Finally, typical uses of IOESs in the measurement of intense E-fields are demonstrated, including application areas such as E-fields with different frequency ranges in high-voltage engineering, simulated nuclear electromagnetic pulse in high-power electromagnetic pulses, and ion-accelerating field in high-energy physics.

## 1. Introduction

### 1.1. The Demand for and Characteristics of Intense E-Field Measurement

**Figure 1.**
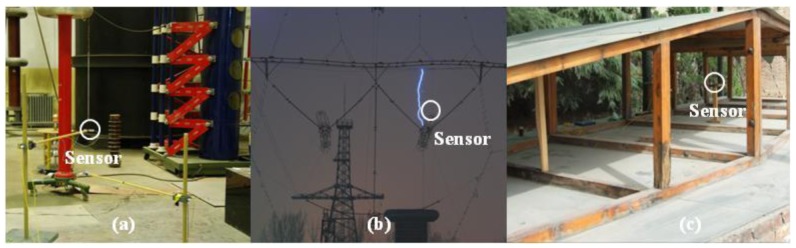
Intense E-field measurement in (**a**) a 1 m rod-plane air gap; (**b**) a window of an ultra-high voltage transmission tower; and (**c**) a NEMP simulator.

The E-field is a fundamental physical parameter that describes electromagnetic phenomena, and measurement of these phenomena is a basic tool for research in various fields of science and technology. First, in high-voltage engineering studies, various phenomena and problems are attributed to the presence of an E-field, e.g., corona discharge, partial discharge, gap breakdown, and the electromagnetic environment. The acquisition of a direct current (DC) field distribution is important in the optimization of a high voltage direct current converter station [1]. An alternating current (AC) power frequency E-field measurement near the surface of an overhead line is essential to the study of electromagnetic environmental problems [2]. Microsecond-order transient E-field detection is necessary for understanding the physical process of long air gap discharge and lightning [3], shown in Figure 1(a) and Figure 1(b). Nanosecond-order transient E-field measurement is significant for the investigation of very fast transient overvoltage (VFTO) in a gas insulation substation (GIS) [4].

Second, high-power electromagnetic pulses (HPEMs) include nuclear electromagnetic pulses (NEMPs), electro-static discharges and high-power microwaves [5]. HPEMs have caused severe damage to life and property in the civil and military domains [6]. The amplitude and frequency characteristics of the HPEM field should be studied to evaluate the hazards associated with HPEMs and to improve the protection of electronic facilities, shown in Figure 1(c).

Third, an important direction in high-energy physics is the development of an ion accelerator. New accelerators such as the Pulse Line Ion Accelerator and Dielectric Wall Accelerator have drawn attention because these devices are highly efficient and have small dimensions [7,8]. The acquisition of new knowledge regarding accelerating E-fields is important for improving insulation performance and acceleration efficiency [9].

Based on the potential applications listed above, the requirements for intense E-field sensors are as follows:

#### (1) Wide Frequency Bandwidth and Large Dynamic Range

For high voltage engineering studies, the E-field has an amplitude range of ∼1 kV/m to ∼1 MV/m and a frequency ranging from DC to several hundred MHz. For HPEMs, the E-field has an amplitude range of ∼1 kV/m to ∼100 kV/m and typically has an ns-order rise time [5]. For an accelerator, the E-field amplitude could be as high as ∼1 MV/m with a 10-ns-order rise time. By contrast, a radio-frequency (RF) field for electromagnetic compatibility (EMC) generally has an amplitude from ∼1 mV/m to ∼1 V/m and a lower cutoff frequency, above 10 kHz [10].

#### (2) High Spatial Resolution and Little Interference to the Original Field

With a frequency range from DC to ∼100 MHz, the corresponding wavelength is ∼1 m to infinity. Therefore, the sensor is usually situated in the near-field area [11]; this placement requires a sensor with small dimensions and a high spatial resolution. In addition, the sensor should contain minimal metal material to guarantee the safety of the power apparatus and reduce the interference to the original field. However, the sensors for EMC testing are often located in the far-field area. A commonly used antenna is suitable for RF field measurements, but it has a low spatial resolution and can severely distort the original field [11].

#### (3) High Stability and Accuracy

Most intense E-field measurement applications occur outdoors. Environmental factors such as temperature variation and vibration should be considered; this consideration requires that the sensor have high stability. Intense E-field measurements must capture both the field distribution and the absolute value, which require high-accuracy measurements. For the RF field test, the sensor is usually located in a stable environment, e.g., inside microwave chamber, and the main concern in this application is the field distribution [12].

### 1.2. The Development of Optical E-Field Sensors

The antennas that are widely used in RF field tests are not appropriate for intense E-field detection. Various types of E-field sensors have been developed based on the modulation of LD or LED [13,14]. Sensors with small dimensions and wide frequency bandwidths have been produced, but these types of sensors are made mostly of metal material, which causes large interferences to the original field.

In contrast, optical sensors based on the electro-optic effect have been widely studied [15,16]. The electro-optic effect refers to the alterations in the optical properties of a medium caused by an imposed E-field, which varies slowly compared with the optical frequency. Optical sensors can be classified into several types according to their specific optical properties.

#### (1) Based on the Change of Absorption Loss

In the **electro-absorption effect**, the absorption coefficient of an optical medium is altered by the E-field such that the optical intensity could be modulated by the E-field [17]. An E-field sensor based on the electro-absorption modulator was developed by Heinzelmann *et al.* with a minimum detectable field of 0.1 V/m and a bandwidth of 6 GHz [18]. This sensor is an active type and is unsuitable for intense E-field measurement.

In the **electro-chromatic effect**, a certain absorption band is generated by an E-field, resulting in a color change of the optical medium [19]. Based on this effect, a fiber E-field sensor was developed by F. Valdivielso *et al.*, in which a single mode fiber was tapered and surrounded by an electro-chromic polymer solution [20]. The response time of this sensor is ∼100 s, which is suitable only for extremely low-frequency field measurement.

#### (2) Based on the Change of Refractive Index

The **Kerr effect**, also known as the quadratic electro-optic effect, refers to changes in the medium refractive index that are proportional to the square of the electric field strength [3]. The Kerr effect exists in any medium with a symmetrical structure. The electro-optic coefficient is small (10^−16^ to 10^−14^ m/V^2^), and therefore, sensors based on the Kerr effect have a low sensitivity and are typically used in the study of E-field distributions in transformer oil [21,22].

The **Pockels effect**, also known as the linear electro-optic effect, refers to changes in the medium refractive index that are proportional to the electric field strength [3]. A sensor based on the Pockels effect has several advantages: it is a passive device that does not need bias voltage and is based on a dielectric material, thus creating little interference to the original field. The modulation of the optical signal produces a wide frequency bandwidth and a fast response. The transmission of optical signals using a fiber can generate electromagnetic immunity and electrical isolation. Optical sensors based on the Pockels effect have been widely studied, and this technical area could be further classified according to whether the sensors are based on bulk optics or integrated optics.

A bulk optic sensor is a common path interferometer based on the birefringence of electro-optic materials and is also known as a Pockels Cell [3]. Since the 1970s, several electric corporations have applied the Pockels Cell to voltage measurement [23,24]. Additionally, the Pockels Cell is also used to detect E-fields [25]. The electro-optic coefficient of a common crystal (e.g., BGO, KDP and LiNbO_3_) is small, and therefore, the Pockels cell has a relatively low sensitivity that is more suitable for intense field detection than EMC field tests.

**Figure 2.**
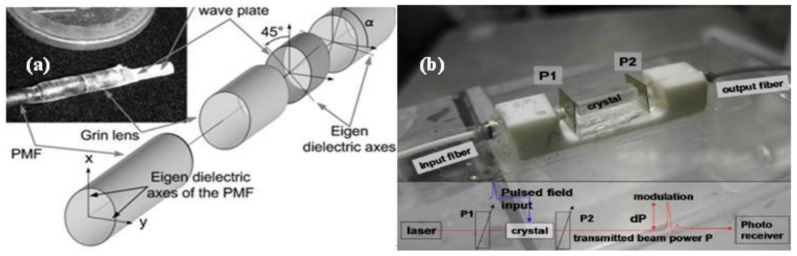
(**a**) Miniature Pockels Cell [34] (Copyright © 2008 OSA, Reprinted with permission); (**b**) Pockels Cell based on KDP crystal [41] (Copyright © 2011 IEEE, Reprinted with permission).

In the initial stages, the Pockels Cell was mainly based on discrete optical components. In 1982, Hidaka studied corona discharge fields using the Pockels Cell and developed sensors for two-dimensional measurement [26–28]. From 1999 to 2007, Ceceja *et al.* applied the Pockels Cell to measurement of the DC E-field and studied the effect of space charge [29–31]. Based on further research developments, discrete components were assembled, which improved the stability and practicality of the sensor, shown in Figure 2. Since 2001, Duvillaret *et al.* have developed assembled Pockels Cells for one-dimensional and two-dimensional detection and demonstrated the simultaneous measurement of E-field and temperature [32–35]. Since 2005, the Naval Research Laboratory (NRL) has comprehensively studied the influence of additional physical effects such as the photo-refractive effect, piezo-electric effect and dielectric property on measurement sensitivity. Various outdoor HPM tests were conducted for solving the problems of low-frequency drift and high-frequency noise [36–41].

A sensor based on bulk optics has prominent advantages: a favorable optical bias can be achieved with a 1/4 wave plate, and the pyro-electric and piezo-electric can be avoided by selecting a non-ferroelectric crystal. Nevertheless, this sensor type also possesses certain disadvantages: the structure of the sensor head is complicated, resulting in lower reliability and higher interference to the original field, and the relatively large dimension of the crystal limits the frequency bandwidth and spatial resolution.

The integrated optical E-field sensor (IOES) has been extensively studied due to the rapid development of integrated optics. In general, a light waveguide is created on the LiNbO_3_ substrate through Ti indiffusion or proton exchange, and the antenna/electrode is fabricated near the waveguide by photolithography [42]. IOESs with various ranges of sensitivity and frequency bandwidth can be achieved by designing specific antennas and electrodes.

The purpose of this paper is to provide a comprehensive overview of the development and application of IOESs for intense E-field measurement and is organized as follows: In Section 2, three types of IOESs are classified and reviewed according to operating principle and sensor configuration. Recent progress and achievements are presented, and the characteristics of different types of IOESs are compared. On this basis, the improvement work of applying IOESs to intense E-field measurement is introduced. Section 3 illustrates the typical applications of intense E-field measurement using IOESs. The measurement of the DC field, power frequency AC field, μs-order transient field, and ns-order transient field are demonstrated for high-voltage engineering. The measurement of simulated NEMP is covered for HPEMs, and for high-energy physics, the measurement of the accelerating E-field of an ion accelerator is presented. Section 4 summarizes the conclusions of this paper and discusses the outlook for future IOES work.

## 2. The Integrated Optical E-Field Sensor

**Figure 3.**
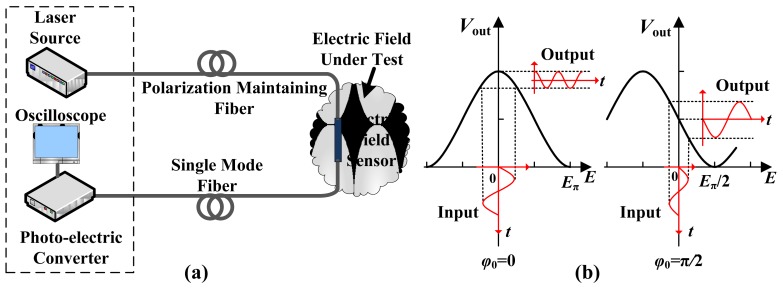
(**a**) Structure of the measurement system; (**b**) Transfer function with different *φ*_0_.

IOESs can be classified into three types according to differences in their waveguide structure and in their operating principle: the Mach-Zehnder Interferometer (MZI), the Coupler Interferometer (CI), and the Common Path Interferometer (CPI). The measurement system is illustrated schematically in Figure 3(a). The linear polarized light generated by the laser source is transported to the sensor through a polarization-maintaining fiber. The optical signal is phase-modulated or polarization-state-modulated by the E-field as it passes through the waveguide. For the MZI or CI types, the phase modulation is transferred to intensity modulation at the output end of the waveguide. For the CPI type, the polarization-state modulation is changed to intensity modulation by the analyzer. The intensity signal is then delivered to the photo-electric converter by a single-mode fiber, where the optical signal is converted into an electrical signal. The sensor is situated in an intense electromagnetic environment, whereas the electronic devices are located in an electromagnetic shielding environment. The effective electrical isolation could be provided by the input/output fiber.

The ordinary Coupler Interferometer has a complicated transfer function [43], while both the MZI and CPI have a simple sinusoidal type [43,44], expressed as:

(1) $$V_{\text{out}}=A\cdot \left[1+b\cdot \cos(\varphi_{0}+\varphi (E))\right]$$

where *A* represents the optical power and photo-electric conversion coefficient; *b* represents the extinction ratio, usually ranging from 0.9 to 1; *φ*_0_ is the operating point, *i.e.*, the phase mismatch under a zero *E*-field; and *φ*(*E*) is the phase mismatch caused by the external E-field. The transfer function is illustrated in Figure 3(b). Ideally, *φ*_0_ = π/2 (also known as the quadrature point), which results in an approximately linear input *E* and output *V*_out_ near this point. The output waveform is severely distorted, with *φ*_0_ deviating significantly from the quadrature point. *φ*(*E*) could be described as:

(2) $$\varphi (E)=\frac{E}{E_{\pi }}\pi$$

where *E*_π_ is the E-field strength that results in a phase modulation equal to π (also shown in Figure 3). *E*_π_ is known as the half-wave E-field and determines the dynamic range of the sensor.

### 2.1. Mach-Zehnder Interferometer

#### 2.1.1. Development of the MZI-Based E-Field Sensor

**Figure 4.**
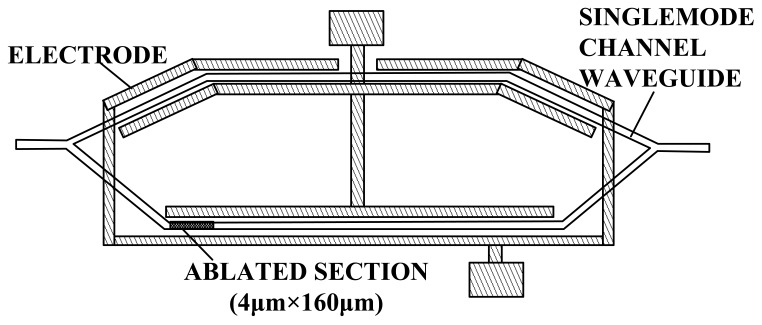
IOES produced by NRL; the optical bias *φ*_B_ is adjusted by laser ablation [51] (Copyright © 1995 IEEE, Reprinted with permission).

The typical structure of an IOES based on the MZI is shown in Figure 4. As the linear polarized light enters the waveguide, it is equally divided by the first Y-branch. The antenna and electrode amplify the external E-field. Next, the refractive index of the two waveguide arms are differently altered, resulting in a phase mismatch between the optical signals in the two arms. The two phase-modulated optical signals interfere with each other when they merge at the second Y branch. Consequently, the two optical signals become an intensity-modulated optical signal. The transfer function of the measurement system is expressed in Equation (1). To improve the sensitivity, the largest electro-optic coefficient *γ*_33_ is adopted, and the X-cut Y-propagating LiNbO_3_ wafer is commonly used. *φ*(*E*) can be described as [45]:

(3) $$\varphi (E)=\frac{2\pi \Gamma {n_{e}}^{3}r_{33}L_{\text{el}}}{\lambda_{0}}E_{\text{in}}$$

where Γ is the electro-optic overlap integral, representing the interaction of the E-field and the optical field; *n*_e_ is the extraordinary refractive index; *L*_el_ is the electrode length; *λ*_0_ is the vacuum wavelength; and *E*_in_ is the E-field imposed on the waveguide in the Z direction.

The LiNbO_3_ material is a ferro-electric crystal with a pyro-electric property. A temperature change induces polarization charge that accumulates on the surfaces perpendicular to the principal axis [46,47]. Therefore, an additional E-field in the Z direction is generated by the pyro-electric effect. The amplitude of this field is estimated to be as high as ∼100 kV/m with a temperature variance of 1 °C [46], resulting in an additional phase mismatch. *φ*_0_ can then be expressed as:

(4) $$\varphi_{0}=\varphi_{\text{B}}+\varphi_{\text{T}}$$

where *φ*_T_ is the phase mismatch caused by temperature, mainly due to the pyro-electric effect, and *φ*_B_ is the optical bias, determined by the intrinsic optical path difference of the two arms.

In the early stages of research in this field, an IOES based on MZI was used for RF tests in the EMC. The main objectives focused on the optimization of the antennas and electrodes to improve the sensitivity and frequency bandwidth. During the period from 1980 to 1995, the MZI-based IOES was first reported and comprehensively studied by Bulmer *et al.* at the NRL [46–51]. The fabricated sensor had a minimum detectable E-field of 1 V/m and a bandwidth of 300 MHz. As shown in Figure 3(b), a value of *φ*_0_∼π/2 is desirable, which requires *φ*_B_∼π/2 and *φ*_T_∼0 (see Equation (4)). To ensure that *φ*_B_∼π/2, the length difference of the two arms should be several micrometers. The errors in the photolithography and waveguide fabrication could not meet this accuracy requirement, leading to an uncontrollable value of *φ*_B_. Laser ablation was proposed to improve the controllability of *φ*_B_ [50,51], as shown in Figure 4.

**Figure 5.**
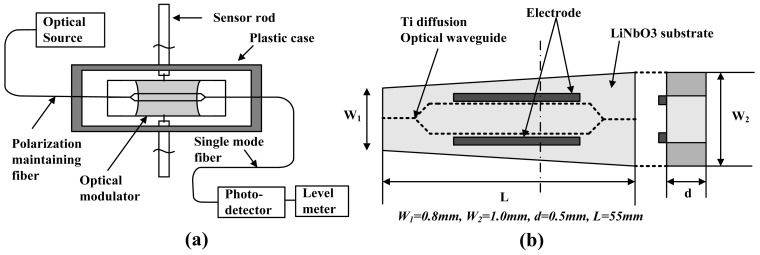
(**a**) Bandwidth improvement using a resistive antenna [53]; (**b**) The piezo-electric resonance is restrained using a trapezoidal substrate [56] (Copyright © 2000 John Wiley & Sons, Inc., Reprinted with permission).

To maintain *φ*_T_∼0, the two surfaces in the Z direction were short-circuited via a conducting paste, and a more favorable temperature stability was obtained [51]. It should be noted that laser ablation requires complicated technology and that the short-circuit of the surfaces could also result in higher interference to the original field. From 1991 to 2002, Tajima *et al.* at the NTT Corp. developed high-sensitivity MZI-based IOESs, as shown in Figure 5(a) [52–58]. The resonance of the dipole antenna limits the high frequency characteristic of the sensor, and a resistive antenna was developed to expand the bandwidth [53]. The measurement accuracy could be altered via the piezo-electric resonance phenomenon of the LiNbO_3_ crystal; in other words, an E-field with a specific frequency stimulates the mechanical resonance of the crystal through the inverse piezo-electric effect, and the resulting strain alters the refractive index through the photo-elastic effect. The LiNbO_3_ substrate was shaped as a trapezoid, dispersing the resonance energy and restraining the piezo-electric resonance phenomena, as shown in Figure 5(b) [56]. To ensure that *φ*_B_∼π/2, a method for adjusting *φ*_B_ via strain was proposed [57]. A three-dimensional sensor was implemented by locating three one-dimensional sensors on the surfaces of a triangular prism; the fabricated sensor had a bandwidth greater than 10 GHz and a minimum detectable field of less than 22 mV/m [58].

**Figure 6.**
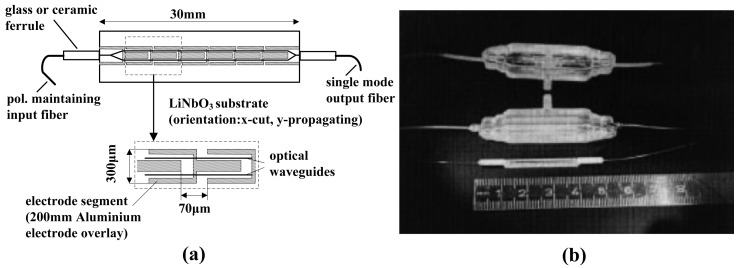
(**a**) Segmented electrodes proposed to improve the sensitivity and bandwidth [59]; (**b**) The sensor sealed with epoxy resin [62] (Copyright © 1997 IEEE, Reprinted with permission).

From 1992 to 1997, Petermann *et al.* at the Tech. Univ. Berlin developed IOESs with segmented modulator electrodes [59–63]. The antenna was located along the waveguide, reducing the equivalent input impedance and improving the match between the dipole antenna and the modulator, as shown in Figure 6(a). The fabricated sensor had a sensitivity of 1 mV/m and a bandwidth of 3 GHz. A sealed sensor was also implemented to detect an E-field in liquid, as shown in Figure 6(b).

**Figure 7.**
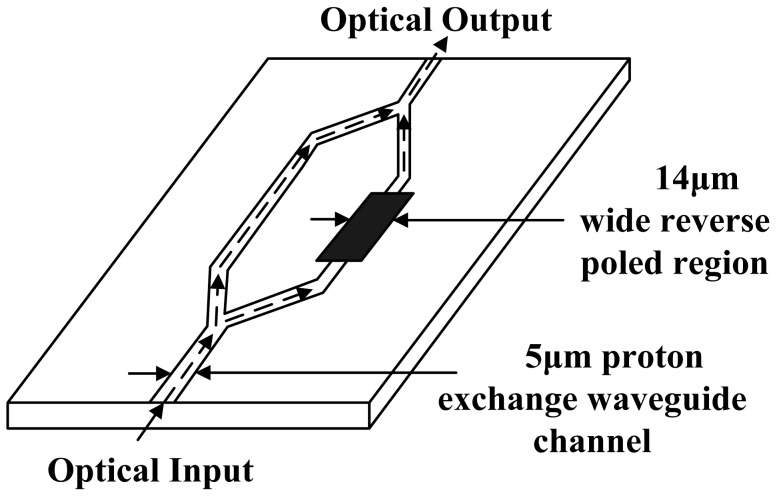
The domain-inversed IOES developed by the Srico Corp [64].

From 1994 to 1995, an MZI-based IOES with neither an antenna nor an electrode was developed separately by the Srico Corp. and the Univ. of British Columbia (UBC) [64,65]. The two arms of the MZI had different Ti-ion concentrations corresponding to a distinct Curie temperature. Under a certain temperature (e.g., 1,150 °C), domain inversion occurs in the area with a higher Ti-ion concentration, and the electro-optic coefficient is reversed. The two arms of the MZI have different electro-optic coefficients. The IOES developed by Srico Corp. had a minimum detectable field of 0.22 V/m and a bandwidth of 1 GHz, as shown in Figure 7.

#### 2.1.2. Improvement of Applying MZI Sensors to Intense E-Field Measurement

As stated above, past researchers focused on the RF field test, usually for amplitudes less than 1 V/m. The widely developed IOES performed well in high-frequency applications, but the low-frequency characteristics (DC to 10 kHz) of the sensor were rarely studied. Almost none of the MZI-based IOESs was designed for intense E-field measurement.

**Figure 8.**
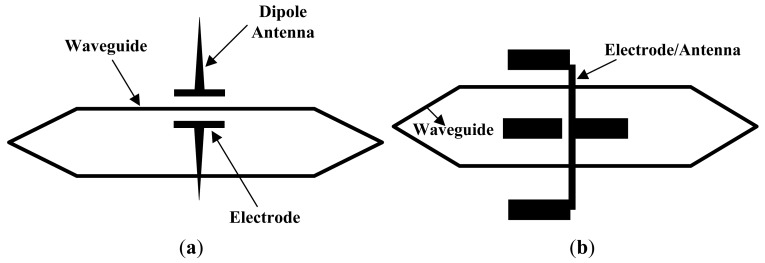
(**a**) IOES with an *E*_π_ of 600 kV/m; (**b**) IOES with an *E*_π_ of 70 kV/m [45].

Based on existing knowledge and the requirements for measuring an intense E-field, the structures of the antenna and electrode were optimized, and IOESs suitable for intense E-field detection were developed by Tsinghua Univ. (THU) [45]. The fabricated sensors have *E*_π_ ranges from ∼10 kV/m to ∼100 kV/m and a flat frequency response curve from the power frequency to greater than 100 MHz. For the design shown in Figure 8(a), two electrodes with a distance of 40 μm were connected to the vertical dipole antennas with a length of 2 mm, and the *E*_π_ was approximately 600 kV/m. For the IOES illustrated in Figure 8(b), dipole antennas were combined with the electrodes. The gap between the electrodes was enlarged to 100 μm, and the length of the antenna was shortened to 10 mm, leading to an *E*_π_ of approximately 70 kV/m.

**Figure 9.**
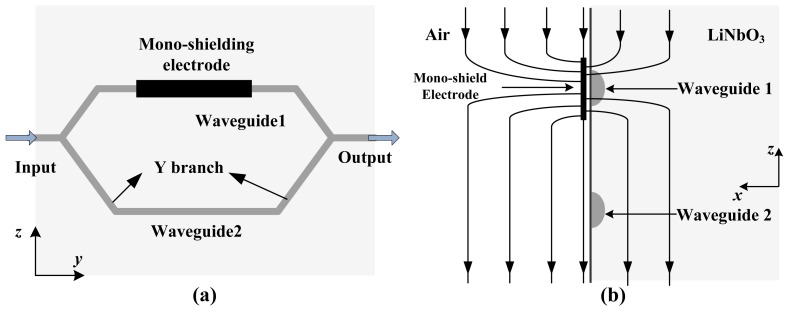
(**a**) Top view of the sensor with a mono-shielding electrode located on one of the waveguide arms; (**b**) Side view of the sensor; the electric field line is altered due to the electrode.

To expand the dynamic range, an IOES with a mono-shielding electrode was developed and is illustrated schematically in Figure 9. The E-field distribution around the waveguide is altered by the mono-shielding electrode, resulting in unbalanced modulation on the two arms of the MZI.

The mono-shielding gold electrode was fabricated by photolithography, with a thickness of 500 nm and a width of 100 μm. The fabricated sensors have *E*_π_ ranges from 2,000 kV/m to 8,000 kV/m; the *E*_π_ is altered by changing the length of the electrode (5 mm to 20 mm) and meets the requirement for air discharge field measurement.

**Figure 10.**
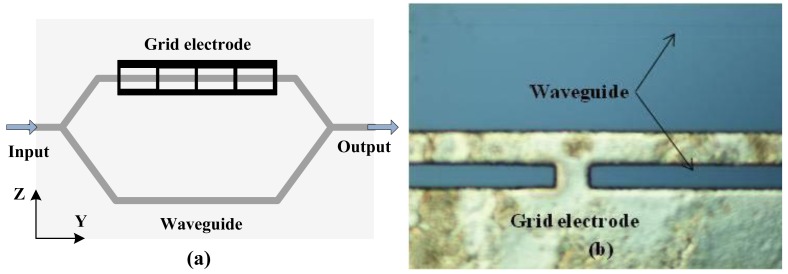
Mono-shielding electrode optimized as a grid type. (**a**) Schematic of the sensor; (**b**) Micrograph of the fabricated electrode.

A buffer layer (Si or SiO_2_) should be added between the waveguide and electrode to eliminate the influence of the metal on the waveguide. However, the buffer layer deteriorates the frequency response [66] and the stability [67]. The mono-shielding electrode was optimized as a grid type, shown in Figure 10 [68]. This improvement ameliorates the stability while maintaining the electrical characteristics of the sensor. The reduction of metal material may also generate less interference to the original field.

The MZI type is the most widely studied IOES; it has a simple geometrical structure with an *E*_π_ ranging from ∼1 V/m to ∼1 MV/m and is tuned using different types of antennas and electrodes. The key problems with this type of IOES are the controllability of *φ*_B_ and the stability of *φ*_0_. The optical bias *φ*_B_ may severely deviate from the designed value due to errors in the photolithography and waveguide fabrication processes, and the operating point *φ*_0_ drifts drastically in response to temperature variations due to the pyro-electric effect. The drift rate is ∼1.25 °/°C for an ordinary MZI sensor and ∼5 °/°C for the domain inverse type [69].

### 2.2. Coupler Interferometer

#### 2.2.1. Development of the CI-Based E-Field Sensor

For MZI-based IOESs, the controllability of *φ*_B_ is improved by laser ablation [50,51] and strain adjustment [57], although both processes add to fabrication complexity. Compared with the MZI, the Coupler Interferometer has a natural quadrature optical bias. The transmission and phase modulation of the optical signal in the waveguide is identical for the MZI and CI. The optical fields in the two arms of the CI couple with each other, thus changing the phase modulation to intensity modulation.

**Figure 11.**
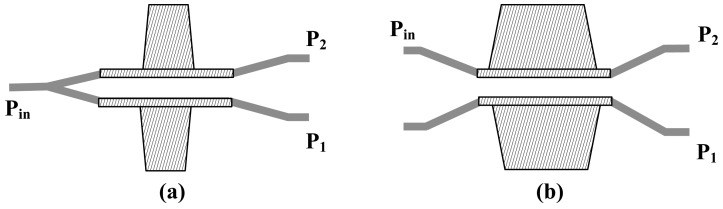
CI-based IOES with three ports and four ports [43].

In 1986, Thaniyavam initially proposed the CI-based modulator. The experimental result is consistent with the theoretical analysis, and both show that the modulator has a quadrature optical bias [70]. In 1988, Howerton *et al.* at the NRL created the CI-based IOES and compared the properties of IOESs based on the MZI and CI [71]. Subsequently, a CI-based IOES with four ports, shown in Figure 11, was reported by Kanda *et al.* [43].

#### 2.2.2. Improvement of Applying CI Sensors to Intense E-Field Measurement

**Figure 12.**
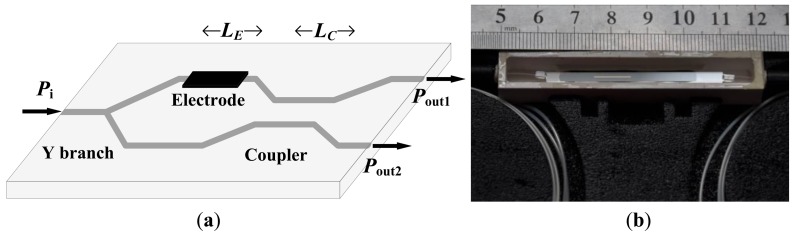
(**a**) Schematic of CI-based IOES; (**b**) Sensor after encapsulation.

A CI-based IOES can improve the controllability of *φ*_B_, but it has a complicated transfer function [70]. A new CI-based IOES has been proposed that has a simple sinusoidal transfer function and a more favorable optical bias; this IOES has the advantages of both the MZI and conventional CI types [72]. The structure of the novel sensor is shown in Figure 12(a). The width of the waveguide was designated to be 6 μm to support a fundamental mode only. The distance of the coupler was selected as 7 μm, and the interaction length *L*_C_ was chosen as 2.8 mm. The fabricated sensor is shown in Figure 12(b) and has dimensions of 7 × 1 × 1 cm^3^.

**Figure 13.**
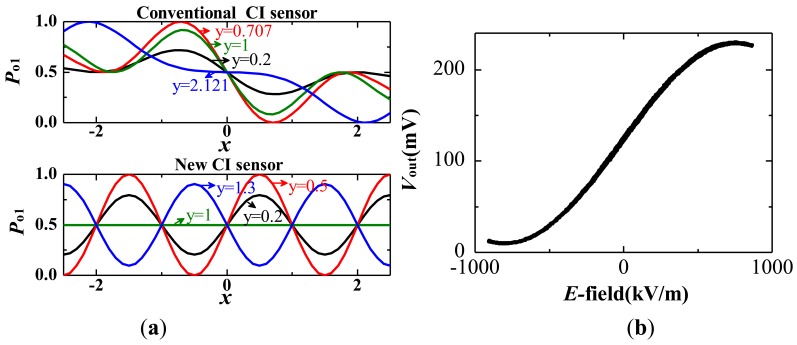
(**a**) Theoretical transfer function of a conventional CI (upper curve) and novel CI (lower curve); (**b**) Experimental transfer function of the novel CI-based IOES.

The theoretical and experimental results of the transfer function are shown in Figure 13, both illustrating that the novel CI sensor has a sinusoid transfer function. Three sensors were fabricated, and their optical biases deviate from the ideal value by 3.3°, 7.9°, and 14.5°. This CI-based IOES has the ability to effectively improve the controllability of *φ*_B_. The modified CI-based IOES has the advantages of the MZI type and improves the controllability of *φ*_B_. However, the problem of the temperature stability of the operating point *φ*_0_ remains unsolved.

### 2.3. Common Path Interferometer

#### 2.3.1. Development of the CPI-based E-Field Sensor

**Figure 14.**
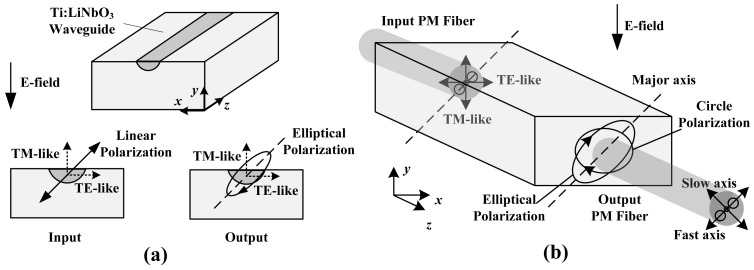
Structure of CPI-based IOES. (**a**) Polarization state of the input and output light; (**b**) Coupling of the fiber and the waveguide.

The potential advantage of the MZI lies in its high sensitivity; thus, the MZI-based IOES has been widely applied to the EMC test. Because the potential advantage of the CPI is high stability, it has drawn much attention in the area of voltage measurement and intense E-field detection. The structure of the CPI is illustrated in Figure 14.

The Ti-indiffusion waveguide is constructed on a Z-propagating LiNbO_3_ substrate. As linear polarized light enters the waveguide, it is divided into a TE mode and a TM mode. Imposing the E-field in the Y direction, *n_x_* and *n_y_* are modified as follows [73]:

(5-1) $$n_{\text{TE}}=n_{x}=n_{\text{o\_TE}}+\frac{1}{2}n_{\text{o}}^{3}\gamma_{22}E_{y}$$

(5-2) $$n_{\text{TM}}=n_{y}=n_{\text{o\_TM}}-\frac{1}{2}n_{\text{o}}^{3}\gamma_{22}E_{y}$$

where *n*_o_ is the ordinary refractive index. The refractive indices *n*_o_TE_ (X direction) and *n*_o_TM_ (Y direction) are slightly different due to the Ti indiffusion, *γ*_22_ is the electro-optic coefficient, and *E_y_* is the E-field imposed on the waveguide in the Y direction.

When emitted from the waveguide, the TE mode and TM mode display a phase difference, and the light is transferred from linear polarization to elliptical polarization. The elliptically polarized light is analyzed by a polarization beam splitter (PBS) or by an analyzer. The transfer function of the measurement system is also presented in Equation (1). Similar to that of the MZI, the phase mismatch of the CPI could be described as *φ* = *φ*(*E*) + *φ*_B_ + *φ*_T_:

(6-1) $$\varphi (E_{y})\approx \frac{2\pi \Gamma {n_{o}}^{3}r_{22}L}{\lambda_{0}}E_{y}$$

(6-2) $$\varphi_{\text{B}}=\frac{2\pi (n_{\text{o\_TE}}-n_{\text{o\_TM}})L}{\lambda_{0}}=\frac{2\pi \Delta n_{\text{o}}L}{\lambda_{0}}$$

where *L* is the waveguide length. (*n*_o_TE_-*n*_o_TM_) is related to the waveguide width, such that *φ*_B_ could be controlled by changing the waveguide width.

Imposing the E-field in the Z direction, *n_x_* and *n_y_* are altered as follows [73]:

(7-1) $${n_{\text{TE}}}^{\prime }=n_{\text{o\_TE}}+\frac{1}{2}n_{\text{o}}^{3}\gamma_{13}E_{z}$$

(7-2) $${n_{\text{TM}}}^{\prime }=n_{\text{o\_TM}}+\frac{1}{2}n_{\text{o}}^{3}\gamma_{13}E_{z}$$

Next:

(8) $$\varphi (E_{z})=\text{O}$$

The additional E-field caused by the pyro-electric effect is along the Z direction. Thus, *φ*_T_ approaches zero in theory, indicating that the CPI-based IOES will not be influenced by the pyro-electric effect and will have better temperature stability.

A comparison of the IOESs shown in Figure 7 and Figure 14 indicates that both are of the all-dielectric type. The *E*_π_ of the MZI type and the CPI type are presented as Equation (9-1) and Equation (9-2), respectively. The E-field in this case is referred to as that imposed on the waveguide:

(9-1) $${E_{\pi 1}}^{\prime }=\frac{\lambda_{0}}{2\Gamma {n_{e}}^{3}r_{33}L}$$

(9-2) $${E_{\pi 2}}^{\prime }=\frac{\lambda_{0}}{2\Gamma {n_{o}}^{3}r_{22}L}$$

The external E-field and that imposed on the waveguide have an approximate relation *E*_ex_ = *εE*_in_, where *ε* is the dielectric constant. Assuming the domain-inversed waveguide length of the MZI is equal to the waveguide length of the CPI, the ratio is *E*_π1_/*E*_π2_ ≈ (*ε*_33_/*ε*_22_) × (*γ*_22_/*γ*_22_) = 0.075 [74]. The CPI-based IOES has an *E*_π_ that is 13 times larger than that of the domain inversed MZI sensor. With *L* = 10 mm and *λ*_0_ = 1,310 nm, the *E*_π_ of CPI is approximately 74 MV/m. Therefore, a notably large *E*_π_ is another feature of the CPI-based IOES.

**Figure 15.**
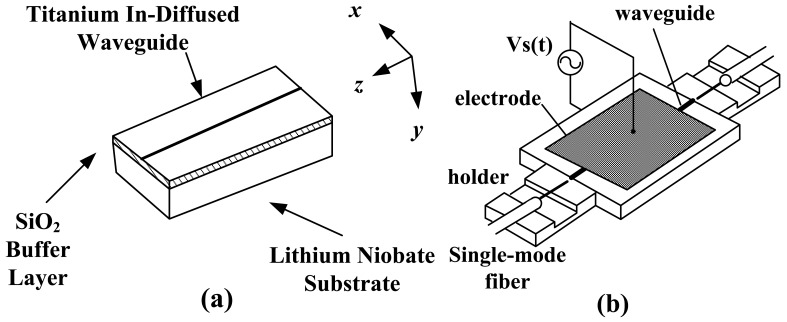
(**a**) Schematic of the CPI-based IOES developed by UBC [77]; (**b**) The CPI sensor developed by the Tokyo Elec. Corp [79] (Copyright © 1997 IEEE, Reprinted with permission).

In 1990, Jaeger *et al.* at UBC initially proposed the CPI-based IOES [75]. The birefringence (*n*_o_TE_-*n*_o_TM_) caused by Ti indiffusion is related to the waveguide width. The waveguide with an optical bias close to π/2 could be obtained by designing waveguides with a series of widths on the same substrate [76]. The relationship between the substrate dimension and the piezo-electric resonance frequency was studied, and it was concluded that *f*_r_ ≈ 6.8 /2*w* MHz (*f*_r_ is the lowest resonance frequency and *w* is the substrate width) [77]. The schematic of the IOES is shown in Figure 15(a), and the E-field sensor was eventually applied to the optical voltage transformer [78].

In 1999, Ogawa *et al.* at the Tokyo Elec. Corp. studied and improved the temperature stability of this type of sensor [79]. The sensor schematic is illustrated in Figure 15(b). The coupling of the fiber and the waveguide was optimized, and the influence of the pyro-electric effect was further reduced by depositing an ITO film on the surfaces of the crystal perpendicular to the principal axis. The fluctuation of *φ*_0_ is as low as ±0.17 rad with the temperature varying from −30 °C to 90 °C.

#### 2.3.2. Improvement of Applying CPI Sensors to Intense E-Field Measurement

**Figure 16.**
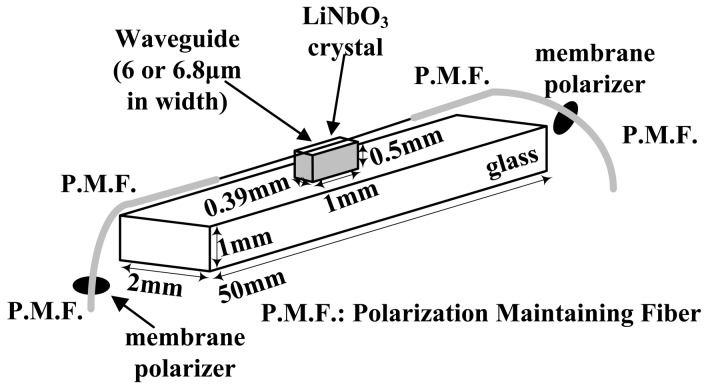
The CPI-based IOES developed by T. Takahashi *et al* [82] (Copyright © 2003 John Wiley & Sons, Inc., Reprinted with permission).

The main objective of UBC and the Tokyo Elec. Corp. was to apply the CPI-based IOES to voltage measurement. Since 1994, Takahashi *et al.* have worked on development of this type of sensor and applied it to discharge field measurement [80–82]. To improve the spatial resolution, a CPI sensor with a small dimension (1 × 0.39 × 0.6 mm^3^) was produced, as shown in Figure 16.

The half-wave E-field of the sensor developed by Takahashi is greater than 100 MV/m. According to the analysis in Section 1, the magnitude of an intense E-field measurement primarily ranges from ∼1 kV/m to ∼1 MV/m. The sensitivity of the CPI-based IOES is so low that it was not able to meet most of the requirements of the applications in measurement of intense E-fields.

**Figure 17.**
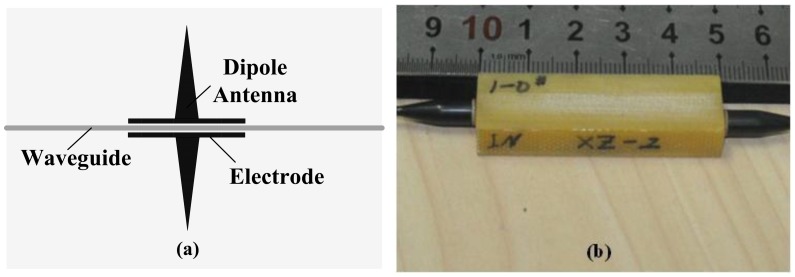
The CPI-based IOES with dipole antenna and electrode. (**a**) Schematic of the sensor; (**b**) Fabricated sensor with dimensions of 5 × 1.2 × 0.5 cm^3^.

To improve the sensitivity, THU designed antennas and electrodes around the waveguide, as shown in Figure 17(a). The Ti-indiffusion waveguide has a length of 2 mm and a width ranging from 10 μm to 12 μm; the antenna has a length of 2 mm, and the electrode has variable dimensions for different *E*_π_. The IOES after encapsulation is shown in Figure 17(b). This type of sensor could be shorter than the MZI or CI types. Because the waveguide with a favorable optical bias in the CPI could have a length on the order of millimeters, the Y branch of the MZI or CI limits the reduction of the waveguide length.

**Figure 18.**
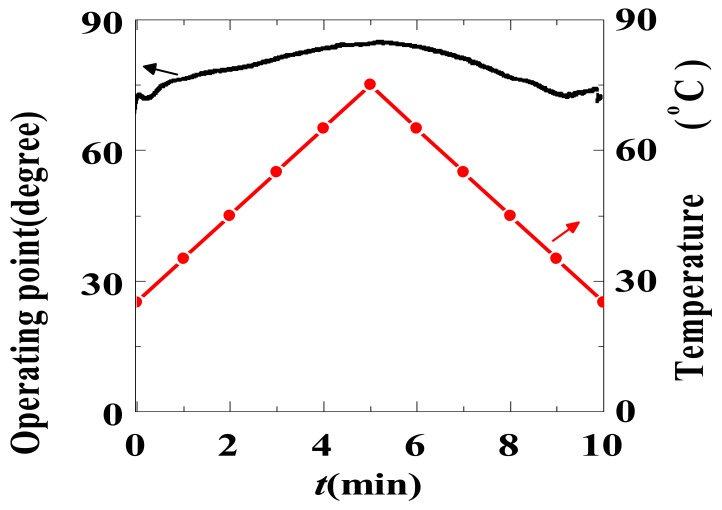
Drift of *φ*_0_ with temperature.

The fabricated sensors have an *E*_π_ of approximately 2,500 kV/m, and the optical biases deviate from the ideal values within 8°, as shown in Table 1. The operating point *φ*_0_ varies slowly with temperature, and the drift rate is approximately 0.015 °/°C/mm (the waveguide length is 20 mm), as shown in Figure 18. This value is similar to the 0.014 °/°C/mm value reported by UBC [68], and both demonstrate that the CPI-based IOES has much better temperature stability.

The CPI-based IOES has overcome the key problems of the MZI or CI types: controllability of the optical bias and temperature stability of the operating point. By designing dipole antennas and electrodes, the dynamic range of the optimized sensor is able to meet the requirement of intense E-field measurement.

### 2.4. Characteristic Comparison of IOES

Comparison of the three types of IOESs yields the following observations:

1. The MZI type is most widely studied. It has a simple geometrical structure, and *E*_π_ ranges from ∼1 V/m to ∼1 MV/m can be realized by varying the design of the antennas and electrodes. The key problem is that the optical bias *φ*_B_ is difficult to control and that the operating point *φ*_0_ is severely affected by temperature.
2. The CI type has a natural quadrature *φ*_B_. The improved sensor has both a better *φ*_B_ and a simple sinusoidal transfer function. Similar to the MZI, the temperature stability of *φ*_0_ remains a challenge.
3. The CPI type could effectively solve the problems that exist in the other two types. The advantages of the CPI type include the following: a *φ*_B_ close to the quadrature point can be obtained by selecting the waveguide width. The pyro-electric effect has no influence on *φ*_0_ in theory, and the sensor has much better temperature stability in practice. However, the disadvantages are that the sensor adopts the smaller electro-optic coefficient *γ*_22_ and the larger dielectric constant *ε*_22_, resulting in a lower sensitivity. An additional polarizer and PBS are needed, thus increasing the complexity of the optical system. The characteristics of the three types of IOESs are summarized in Table 2.

## 3. Typical Applications of Intense E-Field Measurement

### 3.1. E-Field Measurement in High-Voltage Engineering

#### 3.1.1. DC E-Field Measurement

Cecelja *et al.* has studied DC field measurement based on bulk optic sensors [29–31]. The discrete optical components can only be located on the optical bench, which is not realistic for practical measurements. A MZI-based IOES with a mono-shielding electrode was primarily used to measure the DC field without space charge [83].

**Figure 19.**
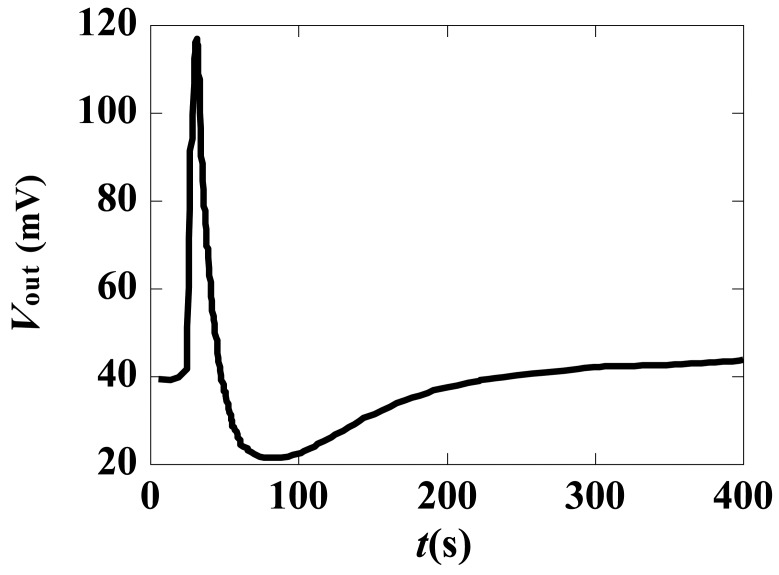
Typical step response of the sensor.

The step response of the sensor is shown in Figure 19. The sensor has a rapidly decaying response due to the dielectric property of the LiNbO_3_ crystal [38]; therefore, the sensor cannot directly measure the DC field.

**Figure 20.**
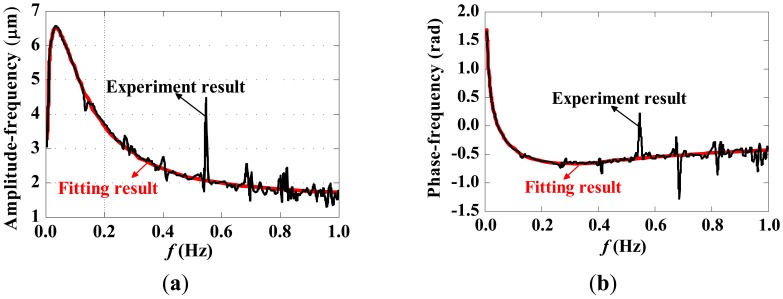
Frequency characteristics and fitting result. (**a**) Amplitude frequency response characteristic; (**b**) Phase frequency response characteristic.

An approximately rectangular pulse was applied to the sensor, and the frequency response of the measurement system was obtained via Fast Fourier Transform (FFT), shown in Figure 20. A vector fitting with three poles and three zeros was performed, and the fitting results are also displayed in Figure 20.

**Figure 21.**
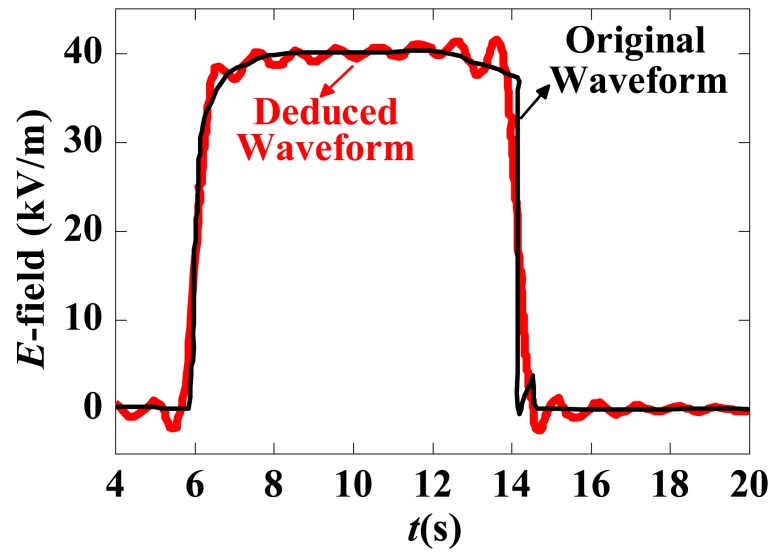
The deduced excitation waveform and the original waveform.

Obtaining the time-domain response under a rectangular pulse E-field together with the frequency response curve of the measurement system, the time-domain input waveform can be restored. The deduced excitation waveform is shown in Figure 21, as is the original excitation waveform; the two waveforms are almost identical.

### 3.1.2. AC Power Frequency E-Field Measurement

**Figure 22.**
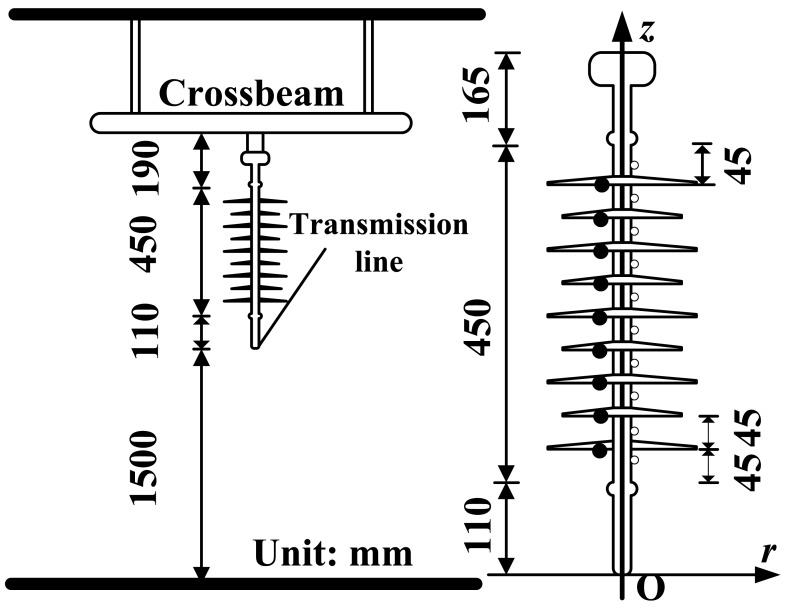
Measurement of E-field distribution along a composite insulator [84] (Copyright © 1997 IEEE, Reprinted with permission).

Measurement of the power frequency E-field distribution along composite insulators is important for the optimization of the insulator structure [84]. The experimental configuration is shown in Figure 22, which simulates the arrangement of suspension insulators on a tower. The E-field sensor was fixed by an insulated bracket to ensure the proper position of the sensor. The output voltage of the power frequency transformer was fixed at 35 kV during the experiment.

The axial component (direction *z* in Figure 22) of the E-field was measured first. The measured positions were selected at the middle of each of two sheds and were numbered from 1 to 10 in sequence from highest to lowest potential, illustrated in Figure 22 as hollow circles. The radial E-field component (direction *r* in Figure 22) was measured as well. The measurement positions were selected just below each shed and were numbered from 1 to 9 in sequence from highest to lowest potential, shown in Figure 22 as solid circles. The surface of the sensor contacted the surface of the shed as closely as possible.

**Figure 23.**
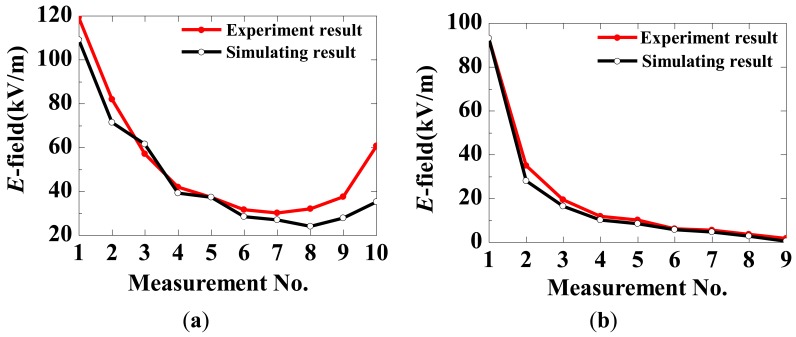
E-field distribution along the insulators with *r* = 30 mm (**a**) in the axial direction and (**b**) in the radial direction.

The measurement and calculation results of the RMS value of the E-field were obtained and are shown in Figure 23 (with *r* = 30 mm). The measured and calculated curves are similar. The distribution of the axial E-field is U-shaped, and the distribution of the radial E-field is monotonously decreased.

#### 3.1.3. Air Gap Discharge E-Field Measurement [85,86]

**Figure 24.**
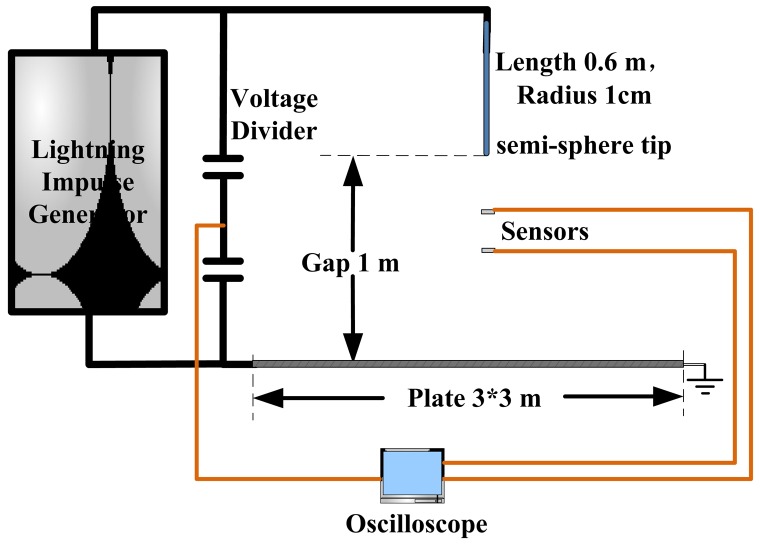
Configuration of a rod-plane air gap discharge experiment [85] (Copyright © 2011 AIP, Reprinted with permission).

The IOES is able to effectively respond to a standard lightning wave (1.2/50 μs) and an EMP with an ns-order rise time [85]. The performance of the adopted sensor is adequate for measuring the E-field in air gap discharges. After calibrating the sensor, a rod-plane discharge experiment under positive lightning impulse was designed and is shown in Figure 24. A rod with a semi-spherical tip with a 1 cm radius was hung at a height of 1 m above a well-grounded plane. The E-field sensors were placed in the gap at distances ranging from 30 cm to 70 cm above the plane.

**Figure 25.**
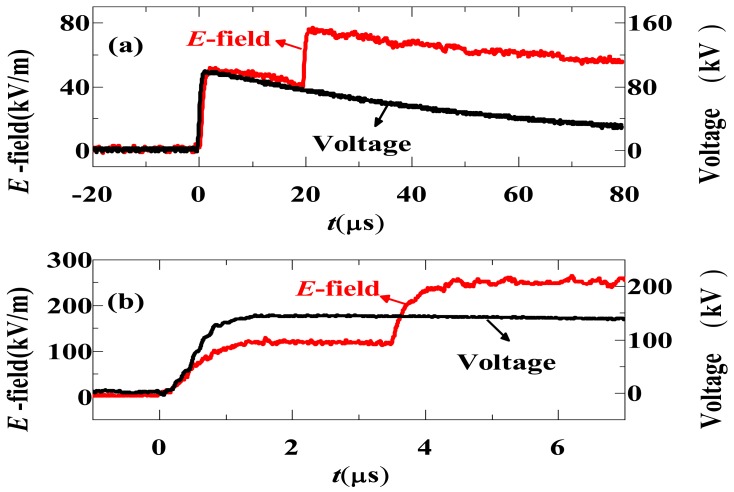
Typical results of E-field at 70 cm above the plane (**a**) with applied voltage of 100 kV and (**b**) with applied voltage of 150 kV [85] (Copyright © 2011 AIP, Reprinted with permission).

Typical results are shown in Figure 25. When the applied voltage is low, the space E-field followed the applied voltage with less than 5% error. When the voltage reached a specific level (e.g., above 200 kV under the experimental conditions), the space E-field was distorted. The E-field strength suddenly increased ΔE, and an E-field step with a rapid rise time was observed. The E-field step was shown to be caused by the space net charge. From the E-step rise time and the corona area range, the average electron drift speed under the experiment situation was estimated to be approximately 0.2 × 10^6^ to 0.6 × 10^6^ m/s.

#### 3.1.4. VFTO E-Field Measurement [87]

Very fast transient overvoltages (VFTOs) are generated by system faults or switching operations in the GIS. The travelling wave has a fundamental frequency of ∼10 MHz due to the compacted structure of the GIS. High-frequency components of ∼100 MHz superposed on the fundamental frequency component are generated due to the refraction and reflection of the travelling wave between different devices.

**Figure 26.**
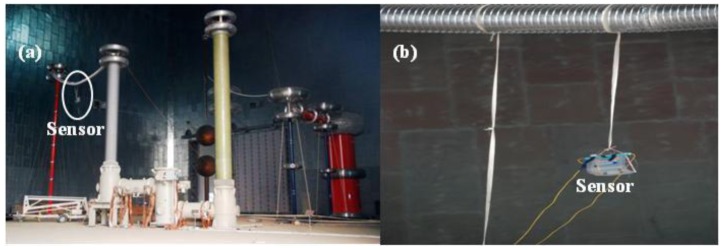
VFTO field measurement: (**a**) Overall configuration; (**b**) Setup of the sensor.

The measuring system developed by THU was applied to a 1,000-kV GIS test setup. Figure 26(a) shows the global view of the equipment: the 1,000-kV experimental transformer is connected to the gas insulated switchgear by the protection resistor, and the output of the switchgear is connected to a capacitive load. The sensor was placed 1 m beneath the center of the long conductor that connects the load and the switchgear, as shown in Figure 26(b).

**Figure 27.**
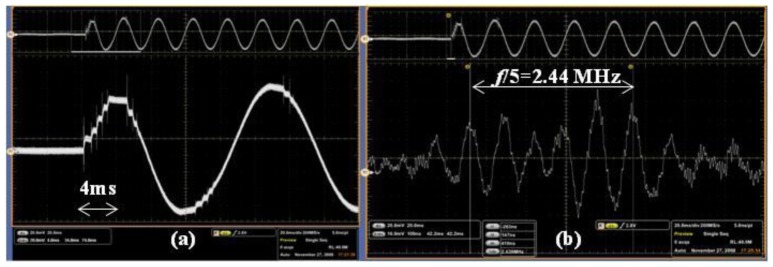
The E-field waveform during a close-operation of disconnector: (**a**) Recording of a power frequency cycle; (**b**) Expansion of (a).

The transient E-field was measured during a close-operation of the disconnector with an RMS AC voltage of 680 kV. As shown in Figure 27(a), the process of the close-operation lasts for approximately one power frequency cycle. With the expansion of Figure 27(a), it was observed that the fundamental frequency is approximately 12 MHz, shown in Figure 27(b). The overvoltage is approximately 1.78 p.u. considering the most serious condition in which the high frequency components are superposed on the peak of the power frequency component.

### 3.2. E-Field Measurement in HPEM

**Figure 28.**
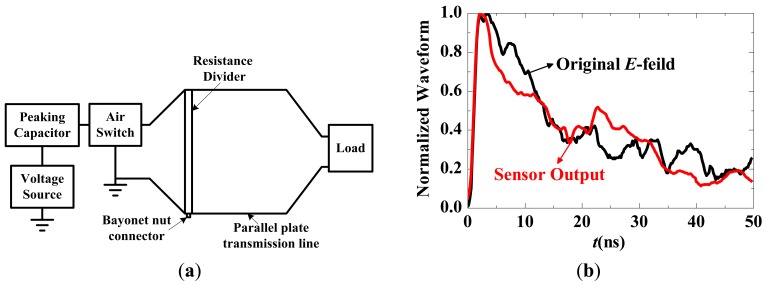
(**a**) NEMP simulator; (**b**) Normalized waveforms of the original E-field and the sensor output.

The nuclear electromagnetic pulse (NEMP) generally has a double exponential pulse shape with a rise time of a few ns, a maximum E-field strength of 50 kV/m, and a full-width half-max time of 200–400 ns [5]. The simulated NEMP can be generated by an NEMP simulator [88] such as a high voltage source, a gas discharge switch and a transverse electromagnetic strip transmission line, as shown in Figure 28(a). The voltage source is connected to the input end of the gas discharge switch through the peaking capacitor. The terminal of the transmission line is a matched load comprising metal film resistors. The voltage U between the transmission lines can be measured by the resistor divider, and the E-field at the central point can be calculated using U/d (where d is the separation of the transmission line). The sensor is located at the central point, and the electronic devices are housed in a screened room.

The simulated NEMP has a rise time of approximately 1.2 ns. The normalized waveforms of the original E-field and the sensor output are shown in Figure 28(b), indicating that the IOES has a response time less than 1.2 ns.

### 3.3. E-Field Measurement in High-Energy Physics

**Figure 29.**
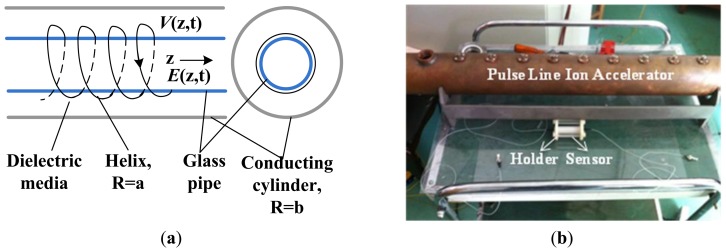
(**a**) Schematic diagram of the PLIA; (**b**) Photo of a PLIA prototype.

A pulse line ion accelerator (PLIA) is a new type of heavy ion accelerator that was developed to meet the need for studying High Energy Density Physics and Warm Dense Matter. Due to the limited space inside the PLIA (cm order), the large E-field strength (kV/m order), and the high frequency (MHz order), measuring the accelerating E-field of the PLIA is difficult [7]. The structure of the PLIA is schematically illustrated in Figure 29(a), and a picture of a PLIA prototype is shown in Figure 29(b). The interior glass pipe has a length of 90 cm and a diameter of 52 mm. The cross-sectional dimension of the IOES is 10 mm, which is suitable for measurement of the PLIA field.

**Figure 30.**
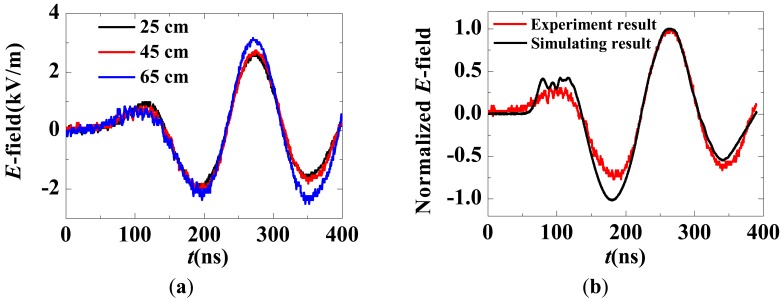
(**a**) E-field along the axis; (**b**) Normalized simulation result and measurement result.

The E-field waveforms on the axis of the PLIA are shown in Figure 30(a). The three measurement points are located at 25 cm, 45 cm and 65 cm away from the front end of the glass pipe. The waveforms appear quite similar in different locations, indicating that the propagation of the electromagnetic field is nearly lossless. With an input voltage of 1 kV, the peak value of the E-field on the axis is approximately 2.9 kV/m. A simulation model of the PLIA was built using the Finite Integration Technique, and the simulated waveform is in agreement with the measured one, as shown in Figure 30(b).

## 5. Conclusions/Outlook

Since the 1980s, the rapid growth of integrated optics technology has led to an increase in worldwide research on integrated optical sensors. Time domain electric and magnetic field measurements are key research topics for scientific work in areas such as high-voltage engineering, high-power electromagnetic pulses, and high-energy physics. Until recently, in these research areas, only integrated optical E-field sensors (IOESs) were able to satisfy most of the application requirements, which include a small size (mm), broadband response (DC ∼ GHz), high signal amplitude (kV/m∼MV/m), and appropriate insulation (MV).

In the search for clear advantages, researchers from the USA, Canada, Japan, Germany and China have focused on the IOES advancements that have been achieved over the past 30 years. These researchers have developed a wide variety of IOES designs, and some have been implemented and applied in practice. As this review details, the Mach-Zehnder interferometer (MZI), coupler interferometer (CI) and common path interferometer (CPI) are the fundamental structures that have been most widely adopted in those designs.

The improvements in applications of IOESs to intense E-field measurement are presented in this work. The theoretical concerns and specific characteristics of these IOESs are illustrated in terms of their structures and key parameters. Finally, selected applications of intense E-field measurement in various research areas are introduced for measuring the time-domain waveform in the nanosecond to microsecond, millisecond, and DC ranges. These applications indicate that IOESs are useful in these applications and will be put into broad use in transient measurement research in areas such as natural lightning physics, gas discharge physics, and HPEMs, among others.

In spite of the prominent advantages and enormous potential of IOESs, challenges still remain for improving IOES performance and expanding their scope of application. First, the operating stability (especially the stability with the temperature variance) should be improved in the future. The mechanism of temperature stability should be studied in-depth, and new materials, innovative structures and design measures should be explored. Second, our experiments and those of others have demonstrated that humidity affects the performance of the sensor; for example, the phase shift between the input and output signals changes with the humidity. Humidity may have additional impacts on the sensor that we have not observed; these impacts should be investigated in detail. Third, the encapsulation of the small sensor should be improved. New packaging materials and techniques should be applied, and the size could be further decreased not only for more accurate measurement but also for reducing the distortion of the original E-field. Fourth, additional work must be conducted for the time-domain and frequency-domain calibration of the intense E-field sensor because it is very difficult to generate a standard field that is sufficiently strong. Finally, questions that are clearly important for on-site applications remain; for example, it remains to be determined how the distortion caused by the sensor in different circumstances should be evaluated and how the accurate original E-field should be obtained. All of the above challenges will motivate continued research in this field.

## Figures and Tables

**Table 1.** Experimental parameters of CPI sensors with antenna and electrode.

**Sensor**	**Optical Bias(degree)**	**Deviation from Ideal Value(degree)**	**Linear Measurement Range(kV/m)**	***E*_π_(kV/m)**
1	98	8	10–500	2,800
2	93	3	10–430	2,180
3	92	2	10–400	2,150

**Table 2.** Characteristics of the three types of IOESs.

**Feature**	**MZI**	**CI**	**CPI**
Controllability of *φ*_B_	Poor	good	good
Temperature Stability of *φ*_0_	Poor	Poor	good
Dimension	small	small	smaller
Sensitivity	high	high	low
Optical system	simple	simple	complex
Frequency Bandwidth	wide	wide	wide
